# Evaluation of the sperm parameters, oxidative stress, and histopathological effects of vitamin B_12_ in preventing *Helicobacter pylori*-induced testicular toxicity: An experimental study

**DOI:** 10.18502/ijrm.v22i5.16439

**Published:** 2024-07-08

**Authors:** Forogh Mohammadi, Maryam Karimi-Dehkordi, Farnaz Pouriayevali

**Affiliations:** ^1^Department of Veterinary, Agriculture Faculty, Kermanshah Branch, Islamic Azad University, Kermanshah, Iran.; ^2^Department of Clinical Sciences, Faculty of Veterinary Medicine, Shahrekord Branch, Islamic Azad University, Shahrekord, Iran.; ^3^Research Institute of Animal Embryo Technology, Shahrekord University, Shahrekord, Iran.

**Keywords:** Helicobacter pylori, Reproductive medicine, Oxidative stress.

## Abstract

**Background:**

*Helicobacter pylori* (*H. pylori*) causes sterility by affecting the reproductive system. Vitamin B_12_ improves sperm quantity and function.

**Objective:**

Vitamin B_12_ protection against *H. pylori* adverse effects was investigated.

**Materials and Methods:**

40 C57 male mice (6 wk) were randomly assigned to 4 equal groups (n = 10) including, group 1 (control without any intervention), group 2 (H), 3 (HP), and 4 (HB) received 1 × 10^9^ colony forming unit (CFU) of *H. pylori*, 1 × 10^9^ CFU of *H. pylori*+phosphate buffered saline, 1 × 10^9^ CFU of *H. pylori+*50 μ g/kg vitamin B ${\;}_{12}$ intraperitoneally, respectively. In the induction groups, the *H. pylori* was orogasterically injected 3 times with 1 cc phosphate buffered saline throughout the day. Then testicular metrics, sperm motility, viability, quantity, and shape, plasma levels of malondialdehyde (MDA), superoxide dismutase, glutathione peroxidase, and total antioxidant capacity were measured. Also, testicular-tissue changes were examined using Johnson scores, tubular differentiation index, and spermatogenesis index. Vitamin B_12_, homocysteine, and testosterone serum levels were examined.

**Results:**

The results showed a significantly lower Johnson score, tubular differentiation index, and spermatogenesis index, and serum level of testosterone and homocysteine as well as a higher MDA level in the H and HP groups than the HB group (p < 0.05). In contrast, the highest superoxide dismutase and glutathione peroxidase enzymes activity and total antioxidant capacity as well as the lowest serum level of MDA were found in the HB group compared to other groups (p < 0.05).

**Conclusion:**

Vitamin B ${\;}_{12}$ increased antioxidant enzyme activity, enhanced sperm parameters, and decreased injury to testicular tissue. It can be used as a potent antioxidant in reducing testicular damage induced by *H. pylori*.

## 1. Introduction 

Healthy reproduction is the foundation for the species' existence and perpetuation. However, there has recently been an increase in accounts of decreasing male fertility (1). According to estimates, infertility impacts millions of reproductive-aged partners globally (2). Male infertility accounts for approximately half of all infertility instances (3). Male infertility can be caused by a variety of factors, including hereditary abnormalities, steroid hormone disorders, hypogonadism, spermatogenesis failure, heavy alcohol consumption, ejaculation disorders, and reproductive infections.

A wide range of microorganisms, such as bacteria, viruses, and parasites, can invade the reproductive organs of men and initiate a cascade of inflammatory reactions that ultimately lead to a decrease in fertility of men (4).

*Helicobacter pylori* *(H. pylori) *is a microaerophilic, gram-negative bacterium with a spiral shape. It thrives in the stomach and triggers a robust cellular and immune system response, leading to chronic gastritis. In combination with bacterial factors, this response contributes to the development of peptic ulcers in 15–20% of infected people and gastric carcinoma in 1–3%, with variations based on the virulence of the infecting organisms and the geographical location (5). There is evidence to suggest that infection with *H. pylori*, particularly those that produce CagA, may have a detrimental impact on human reproductive capacity (6). The relationship between *H. pylori *infection and extra-gastrointestinal illnesses is often attributed to oxidative stress as a potential underlying mechanism. However, there are fewer studies on the effective role of antioxidant supplements in reducing oxidative stress caused by *H. pylori *infection.

Vitamin B_12_ (-[5, 6-dimethylbenzimidazolyl] cobamidcyanide), also known as cobalamin due to the presence of cobalt in its chemical structure, is one of the 8 recognized B vitamins (7). Vitamin B_12_ is essential for spermatogenesis (8). It has been proposed that vitamin B_12_ may affect spermatozoa development. Furthermore, vitamin B_12_ may function as a direct reactive oxygen species (ROS) absorber. There is a growing body of information that suggests a potentially harmful impact of *H. pylori *infection on reproductive health. As a result, this study aims to investigate the effect of *H. pylori *infection on the male reproductive organs and the use of vitamin B_12_ as a protective factor.

Therefore, the effect of *H. pylori *infection and the protective effects of vitamin B_12_ on sperm parameters and testicular tissue are evaluated in this study.

## 2. Materials and Methods

### Experiment and animals

40 healthy male C57 mice (6 wk old, weight 20–25 gr) were purchased from Pasture Institute, Iran. The mice were kept under controlled environmental conditions, with a consistent room temperature of 25 ± 2 C and a humidity range of 40–70%. The mice were housed in plastic cages containing hardwood chips as bedding material, as well as in polypropylene cages that provided a natural 12-hr light/dark cycle. The mice were provided with a standard laboratory pellet meal and were given unrestricted access to water. Mice were randomly divided into 4 groups (n = 10/each).

The first group was without any injection (control group).

The second group was induced with *H. pylori *after an overnight fast and orogasterically inoculated 3 times at a 1-day interval with 1 × 10^9^ CFU *H. pylori *(H group).

The third group was induced with *H. pylori* in their stomach and orogasterically inoculated 3 times at a one-day 1 cc phosphate buffered saline (PBS) (HP group).

The fourth group received *H. pylori *in addition to receiving 50 μ g/kg vitamin B_12_ intraperitoneal injections every week for 3 wk (HB group).

After 1 month, blood samples were taken to confirm the induction of this bacterium in the mice (*H. pylori* > 20). After confirmation of *H. pylori *induction, the samples were collected and the study was ended (9).

### *H. pylori* culture

The *H. pylori *strain ATCC43504 was cultivated in a microaerophilic environment at a temperature of 37 C on *Helicobacter* agar for a duration of 5-7 days. The colonies of *H. pylori *were selected randomly and then submerged in brucella broth that was supplemented with 5–10% fetal bovine serum. This suspension was incubated under microaerophilic circumstances at a temperature of 37 C for 24 hr (10).

### Sample collection

Mice were intraperitoneally anesthetized with ketamine and xylazine, then blood samples were received by heart puncture. Serums were separated from individual blood samples and vitamin B_12_, testosterone, homocysteine, *H. pylori*, total antioxidant capacity (TAC), malondialdehyde (MDA) enzyme, superoxide dismutase enzyme, and glutathione peroxidase enzyme were evaluated. Testicles and epididymis were detached and dissected. Cauda sections of epididymis were incubated in a sperm washing medium (VitaSperm, Inoclon, Iran) supplemented with 10% serum for 40 min at 37 C, and sperms were separated and used for assessment of sperm parameters.

### Histopathological evaluation

The excised testes were immersed in a 10% formalin solution for 24 hr to facilitate fixation. The paraffin slices underwent detachment and processing and were subsequently stained using hematoxylin and eosin staining. The testicles were assessed using tubular differentiation index (TDI), spermatogenesis index (SPI) (11), and modified Johnson scores (Table I) (12). To obtain the SPI, the ratio of active spermatogonia cells to inactive spermatogonia cells was calculated. TDI was obtained, and the percentage of spermatogenic seminiferous tubules or differentiated seminiferous tubules were recorded. 100 seminiferous tubules were analyzed for the evaluation of Johnson score, TDI, and SPI (13).

### Assessment of sperm parameters

The measurement of sperm concentration was conducted using a sperm counting chamber (Sperm Processor located, Aurangabad, India). A volume of 20 μ l of the sample that was obtained was diluted with an equal volume of water. Subsequently, 10 μ l of this diluted mixture was carefully placed into a sperm-counting chamber, allowing for the determination of the number of spermatozoa per milliliter.

The assessment of sperm motility % was conducted using light microscopy at a magnification of × 40. To evaluate sperm movement, a 10 μ l aliquot of the material was placed onto a slide that had been preheated, and the proportion of spermatozoa exhibiting progressive motion was documented. The evaluation of sperm morphology was conducted using the eosin/nigrosin staining method. Initially, 20 µl of washed spermatozoa in PBS was mixed with 40 µl of eosin (Merck, Darmstadt, Germany) for 5 min.

Then, 60 µl of nigrosin (Merck, Darmstadt, Germany) was administered. Smears were produced for each sample, and 200 spermatozoa were counted using a CX31 OLYMPUS light microscope (magnification x100). The proportion of spermatozoa with head, neck, and tail abnormalities and total abnormalities were determined (13).

### Serum analyses

The levels of B12 (vitamin B_12_ II, Roche, Switzerland), testosterone (Testosterone II, Roche, Switzerland), and *H. pylori *in the serum were assessed using the enzyme-linked immunosorbent assay kits (Elecsys 2010 and Cobas e411 analyzers) according to the manufacturer's instructions. The absorbance was measured spectrophotometrically at a wavelength of 450 nm. The determination of homocysteine serum levels was performed using high-performance liquid chromatography.

### Assessment of serum TAC

The TAC of serum was measured using the ferric-reducing antioxidant power method. The basis of this method is the ability of serum to reduce ferric ions Fe ${\;}^{+3}$ to Fe ${\;}^{+2}$ in the presence of tripyridyltriazine (2, 4, 6-Tripyridyl-s-triazineTPTZ) (number 3682–35-7, Sigma, Germany). In this method, the reaction of Fe ${\;}^{+2}$ with TPTZ reagent creates a blue-colored Fe ${\;}^{+2}$ -TPTZ complex with a maximum light absorption property at a wavelength of 593 nm, which can be measured by increasing the concentration of the abovementioned complex by a spectrophotometer (UNICO 2150-UV Spectrophotometer, China) (14).

### Assessment of serum MDA

To measure serum MDA, a working solution containing 0.5 gr of thiobarbituric acid (CAS-number 504–17-6, Merck, Germany) and 80 ml of 20% acetic acid (CAS-number 64–19-7, Merck, Germany) was used. The pH of this solution was adjusted to 3.5 using sodium hydroxide (NaOH) (CAS-number 1310–73-2, Merck, Germany) and its final volume was adjusted to 100 ml by adding 20% acetic acid.

In the next step, 2.5 ml of this solution was poured into a glass test tube along with 100 µl of serum and 100 µl of sodium dodecyl sulfate (1.8%) (CAS-number 151–21-3, Merck, Germany). And the tubes were closed with an aluminum cap. The tubes were placed in a hot water bath for 1 hr and after cooling, they were centrifuged at 4000 rpm (Poland Mpw 260 r). Then, the optical absorption of the supernatant solution was recorded by a spectrophotometer (UNICO 2150-UV Spectrophotometer, China) at a wavelength of 523 nm (15).

### Assessment of serum superoxide dismutase enzyme

To determine the amount of superoxide dismutase enzyme, the Zellbio (ZB-SOD96) kit was used according to the kit protocol. For this purpose, 10 × l of each serum was used and after adding the solutions, its absorbance was measured by a spectrophotometer (UNICO 2150-UV Spectrophotometer, China) at a wavelength of 420 nm.

### Assessment of serum glutathione peroxidase enzyme

To determine the amount of glutathione peroxidase enzyme, both the kit of Zellbio (ZB-GPX96) and the hydrogen peroxide substrate in the kit were used. For this purpose, 10 µl of each serum was used and after adding the solutions, its absorbance was measured by a spectrophotometer (UNICO 2150-UV Spectrophotometer, China) at a wavelength of 412 nm.

**Table 1:** Johnson score


**Level**	**Description**
**10**	Complete spermatogenesis and perfect tubules
**9**	Many spermatozoa present but disorganized spermatogenesis
**8**	Only a few spermatids present
**7**	No spermatozoa but many spermatids present
**6**	Only a few spermatids present
**5**	No spermatozoa or spermatids present but many spermatocytes
**4**	Only a few spermatocytes present
**3**	Only a few spermatogonia present
**2**	No germ cells
**1**	No germ cells or Sertoli cells present

### Ethical considerations

All procedures were confirmed according to the ethical guidelines of the Faculty of Veterinary Medicine, Shahrekord Branch, Islamic Azad University, Shahrekord, Iran, for the Care and Use of Laboratory Animals. This study was approved by the Ethical Committee of Shahrekord Branch, Islamic Azad University, Shahrekord, Iran (Code: IR.IAU.KSH.REC.1402.035).

### Statistical analysis

All the studied data showed normal distribution. Collected data were expressed as the mean ± standard deviation (SD) and p < 0.05 was considered as significant. Where appropriate, ANOVA and independent-sample *t* test were used to define differences within groups. All the analysis was carried out in SPSS software version 22 (SPSS Science, Chicago, IL, USA).

## 3. Result

### Histopathology 

**Figure 1:**
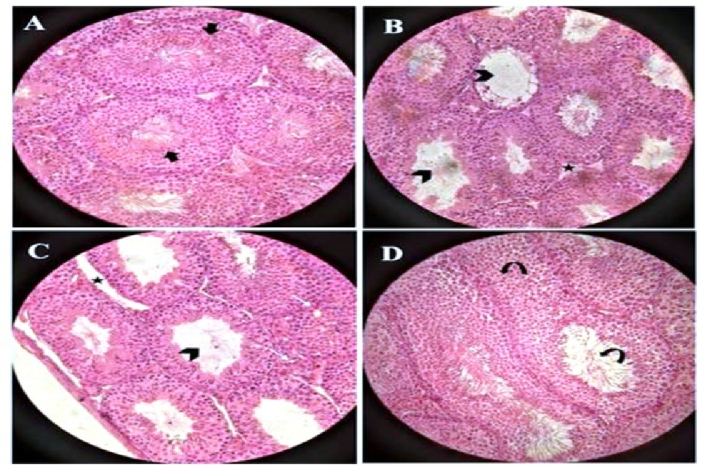
Histopathological evaluations of adult mouse testicular tissue. A) Control, B) Group H, C) Group HP, and D) Group HB. In the control group, seminiferous tubules are arranged normally (black arrow). Abnormal arrangement and diminished seminiferous tubule diameter, vacuolation (white arrow), increased interstitial tissue (star), and decreased sperm density (arrowhead) in the lumens of the H and HP groups. In the vitamin B_12_ + *H. pylori *groups, the seminiferous tubules were arranged normally and contained more sperm. Additionally, the seminiferous tubule walls were thicker (Magnifications: x400).

The testicular tissues of the control group were structurally normal and contained an abundance of sperm cells with normal spermatogonia, layered in multiple layers on the seminiferous tubule (Figure 1A). In the control group, the tubular epithelium was mostly unchanged and comprised Sertoli cells besides spermatocytes and spermatogonia on the basement membrane. Our result showed that infection with *H. pylori *resulted in testicular structural changes characterized by a reduced number of spermatozoa (Figures 1B and C, arrowhead). The seminiferous tubules in the H and HP groups had significant changes compared to the control group, such as seminiferous tubule deterioration and necrosis, reduced interstitial tissue with extensive intracellular spaces, spermatogonia cell loss, and reduction of mature sperm are all indicators of sperm dysfunction.

A significant progress in the HB group was observed, where there was a partial recovery of germinal cells and spermatozoa (rounded arrow) with abundant spermatogenic cell layers (Figure 1D). Leydig cells and typical, weak sperm have been identified in the seminiferous tubules of the vitamin B_12_ treatment group.

### Comparison of histological examinations of mice testis

**Figure 2:**
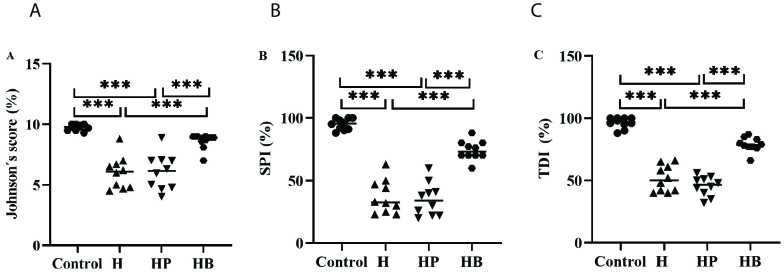
Comparison of testis of mice histological examinations within groups. Comparison of Johnson score, spermatogenesis index (SPI), and tubular differentiation index (TDI) within the control group, mice were induced with *H. pylori *(H group), mice were induced with *H. pylori *and PBS (HP group), and mice received *H. pylori *and vitamin B_12_. ***Shows a significant difference at p = 0.000 between groups.

The testicular levels of Johnson scores, TDI, and SPI exhibited a significant decrease in both the H and HP groups when compared to the control and HB groups (p < 0.001). No statistically significant difference was observed between the control and the HB groups in terms of Johnson scores, TDI, and SPI (p = 0.09, Figure 2).

### Sperm analysis

**Figure 3:**
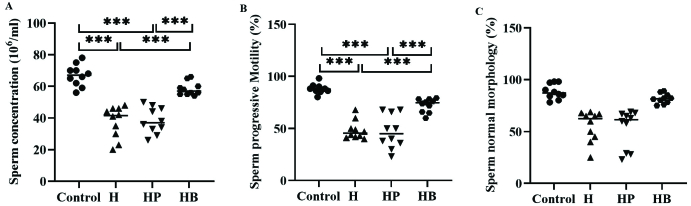
A) Sperm concentration, B) Sperm progressive motility, C) Sperm normal morphology between the control group, H (induced with *H. pylori*), HP (induced with *H. pylori *and PBS) and HB (induced with *H. pylori *and vitamin B_12_) groups. The results are repeated 3 times and the Mean ± SD. ***Show a significant difference at p < 0.001 between groups.

The average values of sperm concentration and progressive sperm motility were found to be considerably lower in the H and HP groups when compared to both the control group and the HB group (p < 0.001). There is no significant variation in the mean value of sperm normal morphology across the various groups (Figure 3).

### Plasma factors

**Figure 4:**
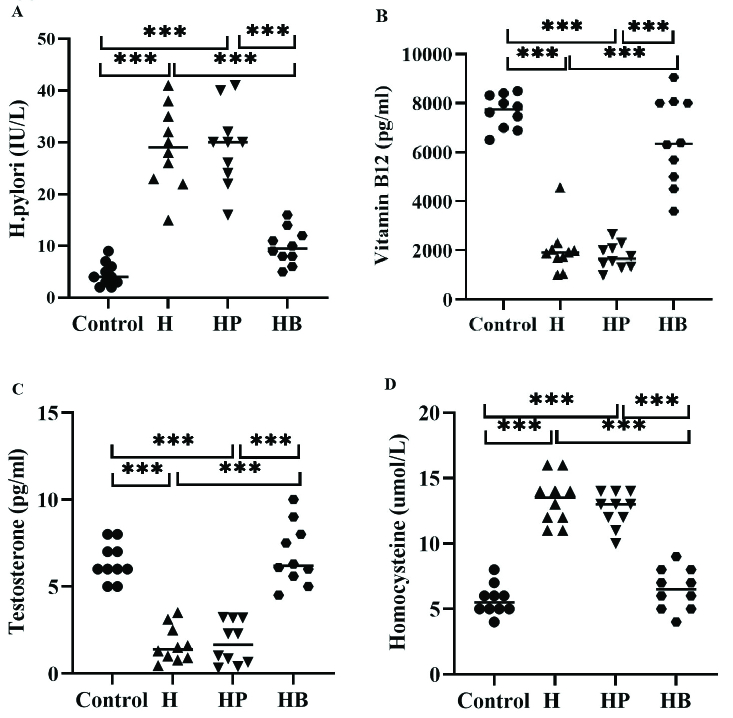
Comparison of *H. pylori*, vitamin B_12_, testosterone hormone, and homocysteine between the control group with H (induced with *H. pylori*), HP (induced with *H. pylori* and PBS), and HB (induced with *H. pylori* and vitamin B_12_) groups. The results are repeated 3 times and Mean ± SD. ***Shows a significant difference at p < 0.001 between groups.

As shown in figure 4, the mean plasma levels of *H. pylori*, vitamin B_12_, testosterone, and homocysteine were significantly decreased in the H and HP groups compared to the control and HB groups (p < 0.001). In addition, no significant difference was observed between the control and HB groups with respect to plasma levels of *H. pylori *(p = 0.178), vitamin B_12_ (p = 0.092), testosterone (p = 0.903), and homocysteine (p = 0.638).

### The effect of *H. pylori* and vitamin B_12_ on MDA level and TAC

**Figure 5:**
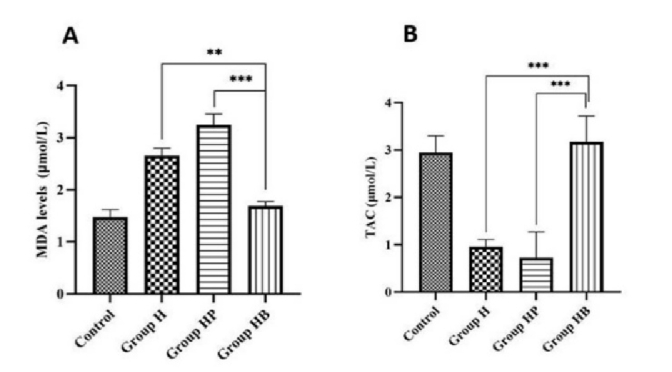
The effect of *H. pylori *infection and vitamin B_12_ on A) Serum MDA level B) TAC. The results are repeated 3 times and the Mean ± SD. **Indicate p < 0.01, ***P < 0.001. H (*H. pylori*), HP (*H. pylori *and PBS), and HB (*H. pylori *and vitamin B_12_)groups. MDA: Malondialdehyde, TAC: Total antioxidant capacity.

In comparison to control group, the concentration of MDA was significantly higher in the H and HP groups (p < 0.001). In the HB group, the amount of MDA was significantly decreased compared to the HP and H groups (p < 0.001). Moreover, based on our findings, *H. pylori *infection decreased TAC relative to the control group (p < 0.001). When compared to the H and HP groups, vitamin B_12_ administration in group 4 substantially averted the decrease in TAC (p < 0.001). Statistical analysis revealed no significant variation between the control and HB groups (Figure 5).

### Effect of *H. pylori* infection on antioxidant enzyme activity in serum

**Figure 6:**
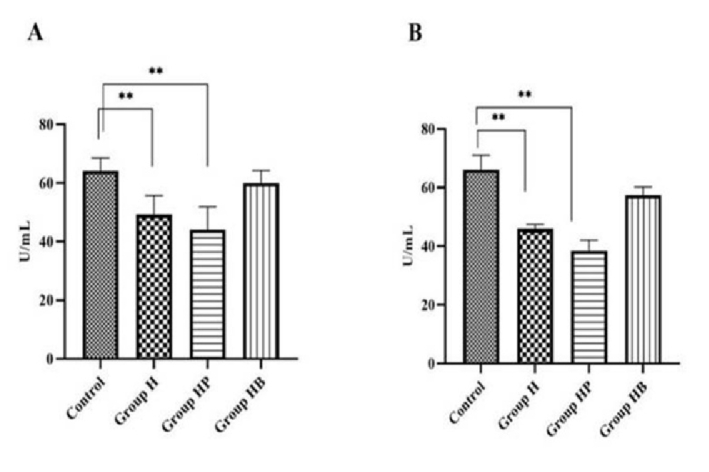
The effect of vitamin B_12_ supplementation on oxidative stress-related enzyme synthesis in infected mice with *H. pylori*. A) The influence of vitamin B_12_ on SOD activity. B) The effects of vitamin B_12_ on GPX activity. Values are presented as the Mean ± SD of 10 animals. **Significantly distinct from control (p < 0.01). H (*H. pylori*), HP (*H. pylori *and PBS), and HB (*H. pylori *and vitamin B_12_) groups.

Superoxide dismutase (SOD) is one of the antioxidant enzymes responsible for preventing ROS production in cells. It converts anion superoxide (O ${\;}_{2}$ ) to low-risk hydrogen peroxide in cells and converts glutathione peroxidase (GPX) and catalase to water. In this study, infection with *H. pylori *significantly decreased SOD levels compared to the control group (p < 0.01). When compared to the H and HP groups, the results showed that vitamin B_12_ had a positive effect on SOD and increased its activity. Compared to the control group, the specific activity of GPX was significantly (p < 0.01) reduced in the H and HP groups. Compared to corresponding controls, pre-treatment with vitamin B_12_ restored this diminished activity (Figure 6).

## 4. Discussion 

Since *H. pylori *infection has been shown to be associated with the histological changes of the seminiferous tubules, the protective effect of vitamin B_12_ against *H. pylori-*induced testicular damage was evaluated in the current study after 3 wk of treatment. The spermatogenesis is progressed nearly over the 4–6 wk in rat testes (16). The decreased Johnson score, TDI, and SPI in the *H. pylori*-infected group confirms the testicular toxicity of *H. pylori *infection. The data obtained from the serum level of SOD, GPX, MDA, and TAC showed that the testicular damage was associated with increased oxidative stress. While, the higher Johnson score, TDI, and SPI in the *H. pylori*-infected + vitamin B_12_ group than in the *H. pylori* group indicated that vitamin B_12_ can reduce testicular damage induced by *H. pylori *and improve the damaged-spermatogenesis*. *Since the vitamin B_12_ treatment group showed higher SOD and GPX enzyme activity and TAC as well as lower MDA levels than H and HB groups, the reduced testicular damage in the treatment group can be contributed to increasing antioxidant capacity by vitamin B_12_.

More than half of the global population is infected with *H. pylori*. In developing nations, the prevalence of infection persists above 80%, while it has radically decreased in developed nations (17). *H. pylori *infection may be linked to health problems outside of the gut system, and it may also contribute to the development of inflammatory diseases. *H. pylori *can induce extra gastric symptoms directly or indirectly via the release of inflammatory mediators and cytokines, molecular mimics, and systemic immune response (18). For many years, research organizations have concentrated on the potential impact of *H. pylori *infections on sperm quality.* H. pylori *infection, particularly by isolates producing the CagA protein, has been suggested as a potential concurrent cause of hypofertility and sperm changes because it has been linked to decreased mobility and a rise in unviable sperms (9). It is clear that *H. pylori *infection reduces sperm motility and viability and has some impact on testicular parameters (19). The exact mechanism by which *H. pylori *may affect sperm parameters is still unknown completely, although involving antigenic mimicry has been hypothesized. Infected individuals may mount a cellular and humoral immune response to bacterial constituents and products that might cross-react with epithelial cells of various organs such as testicular damage (20).

Several studies have examined the protective effects of potent antioxidants (phytochemicals, vitamins, and enzymes) on the testicles of rodents (12, 21–22).

In this study, vitamin B_12_ was selected as it has previously shown to protect against testicular damage caused by cimetidine (23). Vitamin B_12_ has also shown to play a part in the preservation of reproductive processes in the testis. Vitamin B_12_ promotes DNA production, which aids in cell proliferation (24). Rodents deficient in vitamin B_12_ have shown testicular pathological conditions characterized by notable diminishment in testis weight, shrinkage of the seminiferous tubules, and aplasia of spermatids and sperm (23, 25). In oligospermically provoked male rats, for example, methylcobalamin at 1000 µg/kg (6 times a week for 5–10 wk) caused a significant rise in the width of the seminiferous tubules as well as sperm concentration (26). In addition, studies revealed that spermatogenesis would be enhanced when a high dose of vitamin B_12_ is administered to patients with oligospermia in relation to fertility (27). Our results showed that infection with *Helicobacter* leads to a decrease in serum levels of vitamin B_12_.

Low amounts of vitamin B_12_ in the body decrease the enzymatic activity of methionine synthase, which is required to make methionine from homocysteine. This decrease causes a buildup of homocysteine in the bloodstream, also known as hyperhomocysteinemia ( > 15 mol/L) (28). It has been discovered that hyperhomocysteinemia is associated with numerous health issues, including reproductive problems (29).

In this study, hyperhomocysteinemia was observed in groups infected with *H. pylori*. Recent in vitro research uncovered a relationship between sperm parameters such as motility and quantity and thiol concentrations. Such evidence implies a potential homocysteine toxicity to sperm, which may adversely affect sperm parameters due to a depleted vitamin B_12_ level. Hyperhomocysteinemia inhibits nitric oxide synthase pathways in the body, reducing the amount of nitric oxide generated. The enzyme known as nitric oxide synthase exists in spermatozoa and nitric oxide is essential for proper sperm movement, it is plausible that vitamin B_12_ deficiency may impair sperm function via hyperhomocysteinemia-induced nitric oxide deficiency (23). Our results in this study showed that there is a significant relationship between infection with *H. pylori* and the reduction of vitamin B_12_ and the increase of serum homocysteine.

Increased ROS and decreased antioxidant defense lead to redox imbalance, decreased sperm motility, and DNA damage in sperm (30). Due to the abundance of unsaturated fatty acids in their cell membranes, spermatozoa are extremely susceptible to the detrimental effects of ROS (31). Oxidative stress is associated with an increase in the generation of oxygen-free radicals or an unbalance in the redox defense system.

SOD, GPX, and catalase are the 3 major enzymatic components of this system in sperm (32). Our results showed that the activity of SOD and GPX was decreased in the *H. pylori* infected. As the quantity of ROS in the tissue rises, plasma membrane lipids become damaged and peroxidized, and internal components are destroyed, with MDA serving as a measure for lipid peroxidation (33). In our study, the oxidative stress and histopathological effects of vitamin B_12_ pretreatment in the prevention of *H. pylori* injury in mice were evaluated, and it was discovered that vitamin B_12_ supplementation significantly decreased plasma MDA levels and increased GPX and SOD activity.

Along with the results obtained from our research, it was reported that infection with *H. pylori*, especially the CagA strain, can be responsible for infertility in men through a destructive effect on sperm (24).

Furthermore, a study revealed that the presence of *H. pylori *infection leads to elevated levels of inflammatory cytokines in semen. These elevated levels have been associated with a decrease in sperm motility, potential sperm damage, and ultimately a decline in male fertility (7). Finally, this study's findings reported that vitamin B_12_ supplementation has a positive impact on preventing and treating complications of *H. pylori *infection and infertility.

## 5. Conclusion

The results of this study can help to explain that infection with *H. pylori *can lead to infertility by inducing harmful changes in testicular tissue and sperm parameters. In addition, *H. pylori *can cause harm to the reproductive system by destroying the antioxidant balance and decreasing the TAC, and activity of SOD and GPX enzymes. According to the results of the present investigation, vitamin B_12_ is an effective antioxidant that can decrease oxidative stress, enhance sperm parameters, and reduce the rate of testicular tissue damage. The beneficial effects of vitamin B_12_ on sperm parameters can be attributed to improve the efficacy of male reproductive organs, reduced homocysteine toxicity, and decreased ROS accumulation. However, additional research, predominantly clinical, is required to confirm these positive effects.

##  Data availability

Data will be made available on request via email to corresponding authors.

##  Author contributions

Maryam Karimi-Dehkordi designed the study and conducted the research. Maryam Karimi-Dehkordi and Forogh Mohammadi monitored, evaluated, and analyzed the results of the study. Further, Farnaz Pouriayevali reviewed the article. All authors approved the final manuscript and take responsibility for the integrity of the data.

##  Conflict of Interest

The authors declare that there is no conflict of interest.
